# Deciphering the Presence of Active Interscapular Brown Adipose Tissue in Humans

**DOI:** 10.1111/apha.70190

**Published:** 2026-03-18

**Authors:** Joaquin Sanchez‐Gomez, Samuel Ruiz‐Campos, Anabel Chica‐Perez, Andrés Baena‐Raya, Francisco M. Acosta, Christian Wolfrum, Patrick C. N. Rensen, Tania Romacho, Borja Martinez‐Tellez

**Affiliations:** ^1^ Department of Nursing, Physiotherapy and Medicine and SPORT Research Group (CTS‐1024) CIBIS Research Center, University of Almeria Almeria Spain; ^2^ Department of Education, Faculty of Education Sciences and SPORT Research Group (CTS‐1024) CIBIS Research Center, University of Almería Almería Spain; ^3^ Turku PET Centre University of Turku Turku Finland; ^4^ Turku PET Centre Turku University Hospital Turku Finland; ^5^ InFLAMES Research Flagship Center University of Turku Finland; ^6^ Lee Kong Chian School of Medicine Nanyang Technological University Singapore Singapore; ^7^ Department of Medicine, Division of Endocrinology and Einthoven Laboratory for Experimental Vascular Medicine Leiden University Medical Center Leiden the Netherlands; ^8^ Department of Nursing, Physiotherapy and Medicine, Chronic Complications of Diabetes Lab (ChroCoDiL), biomedicine, Integrative Physiology and Therapeutics (BIT; CTS‐1163), CEINSA Research Center University of Almeria Spain; ^9^ CIBER de Fisiopatología de la Obesidad y Nutrición (CIBEROBN) Instituto de Salud Carlos III Granada Spain

**Keywords:** beige fat, brown fat, cardiometabolic health, female, sex, thermoregulation

## Abstract

Brown adipose tissue (BAT) is increasingly recognized as a metabolically active tissue in humans, although its physiological relevance remains incompletely understood. In rodents, BAT is well characterized, with interscapular BAT (iBAT) representing the main thermogenic depot. In contrast, the existence and persistence of iBAT in adult humans have long been overlooked. In this review, we synthesize anatomical, histological, imaging, and molecular evidence supporting the presence of a potentially active iBAT depot within the dorsocervical subcutaneous adipose tissue in humans. Gene expression and histological studies have conclusively identified dorsocervical subcutaneous adipose tissue as iBAT in human neonates. In adults, the persistence of this depot has been suggested by early histological observations, although definitive molecular confirmation is still lacking. More recent data from HIV‐1‐infected individuals report increased expression of BAT‐related markers in the dorsocervical region; however, histological analyses have not consistently confirmed the presence of iBAT in this population. In parallel, two independent cold‐induced ^18^F‐FDG‐PET/CT studies have reported elevated glucose uptake in this area, with a higher prevalence in women. Taken together, these findings suggest that a dorsocervical subcutaneous adipose depot with BAT‐like characteristics may persist into adulthood, particularly in women. Nevertheless, targeted biopsy studies combined with molecular and cellular analyses, together with advanced PET‐CT imaging using tracers capable of assessing thermogenic activity in vivo, are required to clarify whether this tissue represents classical BAT, a beige adipose depot, or a developmentally retained adipose niche. Defining the identity and function of this depot would advance current concepts of human adipose tissue heterogeneity.

## 1 Introduction

Brown adipose tissue (BAT) is a specialized thermogenic fat depot that plays an important role in whole‐body energy expenditure and metabolic regulation by dissipating energy as heat rather than storing it [1]. This thermogenic capacity has classically been attributed to uncoupling protein 1 (UCP1)‐mediated mitochondrial respiration [2]. However, although thermogenic adipocytes were initially defined by UCP1 expression, growing evidence indicates that additional UCP1‐independent mechanisms, such as creatine‐driven substrate cycling, calcium cycling, and lipid turnover, also contribute to heat production [3]. These thermogenic processes are present in distinct adipocyte populations, including classical brown adipocytes, which arise from a myogenic lineage, and beige or brite adipocytes, which emerge within white adipose tissue (WAT) depots in response to specific environmental or physiological stimuli [4]. Beige adipocytes can acquire a brown‐like phenotype, highlighting the marked heterogeneity and plasticity of adipose tissue depots. Both brown and beige adipocytes are present in adult humans. However, their relative contribution to whole‐body metabolism and metabolic health remains incompletely understood. In humans, much of the current knowledge on BAT distribution and activity derives from imaging‐based studies, which have shaped our understanding of BAT anatomy in adulthood.

Human BAT research field gained significant visibility in 2009, when three seminal studies were published simultaneously in The New England Journal of Medicine [5, 6, 7]. Using ^18^F‐fluorodeoxyglucose positron emission tomography‐computed tomography scan (^18^F‐FDG PET‐CT) and molecular confirmation, these studies demonstrated that BAT is present and metabolically responsive to cold exposure in adult humans. Active BAT was primarily identified in the supraclavicular and paravertebral regions, among other anatomical sites [8]. Notably, interscapular BAT (iBAT) was not reported, indicating that BAT detection in adults has been largely restricted to depots identifiable by cold‐stimulated ^18^F‐FDG PET‐CT scans. To date, iBAT has been conclusively demonstrated only in human neonates [9]. Because the use of ^18^F FDG PET‐CT scans in individuals under 18 years of age is ethically restricted due to radiation exposure, our understanding of how BAT prevalence and activity evolve from birth through adolescence remains limited.

Together, these observations raise the possibility that iBAT may exist in adults but remain underdetected using ^18^F‐FDG PET‐CT scans. Therefore, in this review, we aimed to compile scientific evidence supporting the existence of a potentially active iBAT depot within the subcutaneous adipose tissue of the dorsocervical region in humans.

## 2 Interscapular Brown Adipose Tissue in Adults; Historical Evidence and Unresolved Questions

BAT is present in significant amounts in both embryonic and adult mice [10]. While the exact quantity and anatomical distribution of BAT during embryonic development remain incompletely characterized, studies in adult mice have provided detailed insights into its localization. The most predominant BAT depot is situated in the dorsal region, specifically between the *scapulae* (iBAT) and the subscapular area (subscapular BAT). Additionally, rodents exhibit other BAT depots, including cervical BAT, located deep in the dorsocervical region between the scapula and the head; mediastinal BAT, surrounding the aorta within the thoracic cavity; and perirenal BAT, distributed around the kidneys [10]. Although the relative mass of this tissue is similar in mice and humans (1% of total body volume) [11], current evidence suggests that its metabolic activation may have a greater impact on cardiometabolic health in mice than in humans, indicating marked species‐specific differences.

**FIGURE 1:**
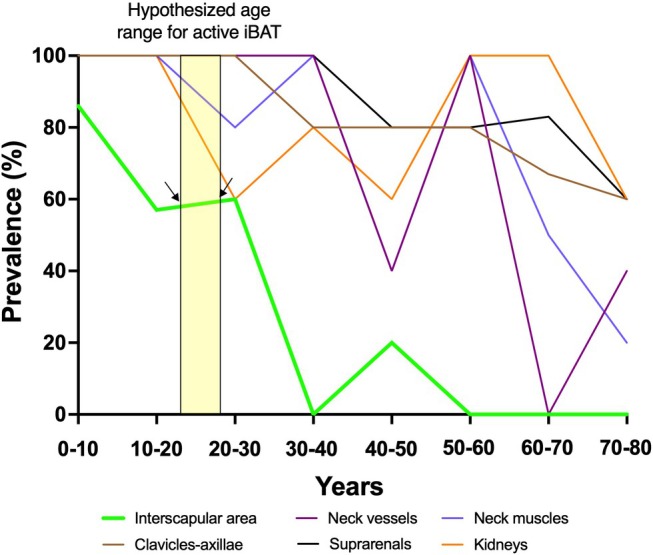
Prevalence distribution of brown adipose tissue (BAT) depots based on the data from 52 corpses at different ages. The data was calculated and extracted from Heaton's 1972 study [13]. The yellow vertical box indicates the adult age range in which we hypothesize metabolically active interscapular BAT (iBAT) may be present. Heaton obtained adipose tissue samples from multiple anatomical regions. In this figure, we highlight the interscapular region, clavicular–axillary areas, neck vessels, neck muscles, kidneys, and suprarenal regions to provide a representative overview of the sampling sites included in her study.

Interestingly, unlike in many mammals, the presence and persistence of iBAT in adult humans remain poorly characterized. Although it is widely assumed that iBAT is present at birth and gradually declines with age, this notion lacks strong empirical validation. While studies using ^18^F‐FDG PET‐CT imaging have demonstrated age‐related reductions in BAT activity [12], most data are derived from middle‐aged adults due to ethical constraints on studies in children. As a result, the developmental trajectory of iBAT from infancy through early adulthood remains unclear. In 1972, Juliet M. Heaton from the Trinity College of Dublin published an important study, some of whose findings were overlooked [13]. She was inspired by different studies performed on corpses of newborns where they described that: *“an interscapular mass lies in a thin diamond‐shaped sheet between the shoulder blades* [14, 15, 16], *known as iBAT*”. Inspired by those studies, she investigated how the presence of BAT varies from infancy to late adult life in healthy individuals who had died suddenly. She collected adipose tissue samples from 18 different body regions in 52 individuals aged 0 to 80 years and grouped the data by decade (e.g., 0–10 years, 10–20 years, and so on). She observed that the prevalence of supraclavicular BAT showed little to no decline with age, a finding that contrasts with data from ^18^F‐FDG PET‐CT studies, which consistently demonstrate an age‐related decrease in this depot [6] (see Figure 1). This discrepancy may be partly explained by the dependence of ^18^F‐FDG‐derived BAT detection on insulin sensitivity, which is known to decline with age [17]. In contrast, iBAT tissue was consistently observed in the dorsocervical region throughout the first three decades of life (i.e., 0–10, 10–20, and 20–30 years). Notably, the prevalence of human iBAT declined sharply after the third decade. Importantly, Heaton also performed histological analyses of the iBAT. Adipose tissue samples were collected bilaterally, fixed in formol saline, paraffin‐embedded, and stained with hematoxylin and eosin, with multiple tissue levels analyzed to ensure representative sampling. iBAT in adolescents and young adults was characterized by adipocytes exhibiting moderate to high lipid content, with a morphology spanning multilocular and unilocular phenotypes. In summary, she reported that iBAT was present in 60% of the corpses she examined from adults aged 20 to 30 years, indicating that iBAT is present in the early stages of adulthood (Figure 1). However, a major limitation of this study is that BAT identification relied on subjective grading of adipocyte multilocularity, a feature that alone is insufficient to define BAT, underscoring the need for cautious interpretation of these findings. Notably, UCP1 could not be assessed, as UCP1 was identified several years after the study was conducted [18]. Collectively, these observations support the possibility that iBAT may persist in the dorsocervical region into early adulthood, but she did not provide evidence for a meaningful thermogenic or metabolic contribution in healthy adults.

Despite the anatomical and histological evidence provided by Heaton [13] and other colleagues [14, 15, 16], subsequent studies in adults have largely failed to directly confirm the presence of metabolically active iBAT using ^18^F‐FDG PET‐CT imaging, largely because most studies have been conducted in middle‐aged males or in patients with at least one cardiometabolic condition. However, it was not until 2013 that Lidell et al. confirmed that UCP1 expression in iBAT obtained from the dorsocervical area of newborn corpses was comparable to UCP1 expression in BAT from the supraclavicular region of healthy adults [9]. Additionally, the gene expression of *ZIC1* and *TBX1* was higher in iBAT than in supraclavicular BAT in adults. In rodents, *ZIC1* is a key marker used to distinguish iBAT from other BAT depots [19], and this appears to be the case in newborns as well. Using magnetic resonance imaging (MRI), the authors also identified iBAT as a thin, diamond‐shaped mass located between the shoulder blades. Histological analyses further confirmed that neonatal iBAT tissue was composed predominantly of brown adipocytes. However, molecular evidence confirming the presence of metabolically active iBAT in the dorsocervical region of adults remains lacking.

Interestingly, certain pathological conditions, such as HIV‐1‐infected individuals, have been linked to abnormal adipose tissue distribution, including the accumulation of adipose tissue in the dorsocervical region. Individuals with HIV‐1 infection are commonly treated with highly active antiretroviral therapy (HAART), which has significantly improved both life expectancy and quality of life [20]. However, this treatment is associated with numerous adverse effects, including HAART‐associated lipodystrophy syndrome (HALS). A hallmark of HALS is abnormal adipose tissue distribution, with patients frequently exhibiting subcutaneous lipoatrophy in the face, arms, and buttocks. Less commonly, HAART‐treated individuals develop lipomatous alterations, the most prevalent being dorsocervical subcutaneous adipose tissue accumulation, commonly referred to as a “buffalo hump”. In this context, Cereijo et al. conducted subcutaneous adipose tissue biopsies in HIV‐1‐infected patients, comparing lipomas from the dorsocervical region (i.e., buffalo hump) with lipomas from other anatomical locations requiring surgical removal (e.g., two pubic, two submaxillary, one arm, and three abdominal) in eight HAART‐treated patients [21]. Additionally, they analyzed subcutaneous adipose tissue samples from 10 age‐matched healthy controls, obtained during minor dermatological procedures, from anatomical sites comparable to those in the lipomatous samples (i.e., abdominal and dorsocervical areas). Their findings revealed that subcutaneous adipose tissue from the dorsocervical area, where iBAT is located, exhibited significantly higher expression of *ZIC1*, *UCP1*, and *PGC1α*, classical BAT markers, as compared to both healthy controls and lipomas from other anatomical regions. These results suggest that the buffalo hump observed in HIV‐1‐infected patients may be enriched in brown adipocyte‐like cells. However, it is important to note that UCP1 expression does not necessarily indicate active thermogenesis, as the two can be dissociated; therefore, the thermogenic activity of the dorsocervical “buffalo hump” adipose tissue should be confirmed using advanced functional imaging techniques [22, 23]. Thus, it is likely that a specific alteration in this adipocyte subtype, induced by HIV‐1 infection and/or antiretroviral therapy, contributes to the pathophysiology of buffalo hump lipomatosis. In contrast, ZIC1 and UCP1 expression were not detected in subcutaneous adipose tissue from the dorsocervical region of the healthy control group (mean age 57.4 ± 3.6 years). This absence supports the hypothesis that metabolically active iBAT is largely restricted to early adulthood and is consistent with Heaton's findings, which reported a 0% prevalence of iBAT in individuals aged 50–80 years (Figure 1) [13].

In addition to these observations, other studies in pathological contexts have provided complementary, sometimes contrasting, evidence regarding the biological nature of dorsocervical adipose tissue. A case report described that facial fat hypertrophy can occur after autologous transplantation using tissue harvested from a “buffalo hump”, and that the transplanted fat retained a partial BAT‐like molecular signature [24]. Consistent with the possibility of altered thermogenic‐related pathways in this depot, increased deiodinase 2 (DIO2) expression has been reported in dorsocervical fat from patients with HIV‐associated lipohypertrophy, although prior assessments using UCP1 expression and thermoneutral ^18^F‐FDG PET‐CT scans have been inconclusive regarding BAT activity [25]. Importantly, BAT‐like molecular or histological features in enlarged cervical depots are not exclusive to HIV‐related conditions; patients with Lamin A/C (LMNA) mutations display enlarged cervical adipose tissue characterized by non‐inflammatory fibrosis and a BAT‐like dystrophy [26]. Conversely, in antiretroviral therapy–associated lipodystrophy, dorsocervical adipose tissue exhibits negligible UCP1 mRNA expression, and histological analyses do not support the presence of BAT compared with abdominal subcutaneous adipose tissue [27]. Taken together, the available evidence suggests that dorsocervical adipose tissue accumulation in these pathological settings may involve heterogeneous adipocyte phenotypes and remodeling processes, with variable expression of thermogenic markers depending on disease context, sampling, and tissue characteristics. Overall, evidence from developmental studies, molecular profiling, and pathological conditions converges on the dorsocervical region as a site with latent brown adipocyte potential. Whether this reflects the persistence of iBAT in healthy young adults, and what its physiological function may be, remain open and important questions.

## 3 Cold‐Induced Glucose Uptake in the Dorsocervical Region: Is Interscapular Brown Adipose Tissue Present?

In 2013, the NUTRIM research group published a narrative review discussing energy dissipation in BAT [28]. In the caption of one figure, they presented a rare example of what they called “iBAT in a lean adult human” as described by the authors. This conclusion was made since a rare glucose uptake by a fat depot in the scapular area was observed in a cold‐induced ^18^F‐FDG‐PET‐CT scan. In 2018, a Finnish group led by Kirsi Virtanen reported that postprandial conditions increased oxygen consumption and blood flow in posterior subcutaneous white adipose tissue at the neck, whereas cold exposure did not induce significant changes in a cohort predominantly composed of women (75%; *n* = 12; age: 35.4 ± 11.3 years; BMI: 27.3 ± 3.9 kg/m^2^). As this anatomical area may be susceptible to imaging artifacts due to tissue compression, the investigators also evaluated subcutaneous adipose tissue at the arm level; however, both oxygen consumption and blood flow were markedly lower in arm subcutaneous adipose tissue compared with posterior subcutaneous adipose tissue at the neck level. Tissue perfusion and oxidative metabolism were assessed by measuring blood flow and oxygen consumption using [^15^O]H_2_O and [^15^O]O_2_ PET tracers, respectively. These findings indicate that thermogenic activity may differ across subcutaneous adipose tissue depots [29]. Subsequently, in 2017, we conducted a randomized controlled trial including 145 healthy young adults, in which cold‐exposed ^18^F‐FDG PET‐CT scans were performed before and after 24 weeks of an exercise intervention [30]. In the baseline ^18^F‐FDG PET‐CT scans, we quantified supraclavicular BAT volume and activity in 133 healthy adults (age: 22 ± 2 years old; BMI: 25 ± 5 kg/m^2^) [30]. Unexpectedly, during the quantification of classical BAT depots, we identified high ^18^F‐FDG uptake in the subcutaneous adipose tissue of the dorsocervical area in adults. We found that in 23 out of these 133 participants (17%), an increased glucose uptake in the dorsocervical area meets the ^18^F‐FDG PET‐CT scan criteria to be considered BAT [31]. Following this, we characterized these 23 individuals and found that they were slightly younger (21 ± 2 years old vs. 22 ± 2 years old, *p* = 0.091) and had a lower BMI (23.2 ± 3.9 kg/m^2^ vs. 25.3 ± 4.8 kg/m^2^, *p* = 0.043) in comparison to the group of 107 individuals who did not exhibit increased glucose uptake in this area. Notably, most individuals showing increased glucose uptake in the dorsocervical region were women (96%; 22 out of 23 individuals) [32]. We also found that cold‐induced ^18^F‐FDG uptake in the dorsocervical region was higher in individuals living with overweight without cardiometabolic complications (MHOO phenotype) compared with individuals living with overweight or obesity with cardiometabolic complications (MUOO phenotype) [33]. To further explore this observation, we exposed another subset of participants to cold using our personalized cooling protocol and observed that dorsocervical and supraclavicular skin temperatures did not decrease in response to the cold stimulus [32]. Interestingly, the warm area over the dorsocervical region displayed a diamond‐shaped pattern, resembling the iBAT distribution described by Heaton in adults [13] and Lidell in neonates [9].

After publishing our finding [32], the National Institute of Diabetes and Digestive and Kidney Diseases published a similar study confirming our observations [34]. In that study, BAT activity was assessed in young adults using cold‐induced ^18^F‐FDG PET‐CT scans, and glucose uptake in the dorsocervical region was likewise reported, occurring more frequently in women than in men (6 of 12 women vs. 1 of 12 men). Although both studies were conducted in relatively young cohorts, several differences merit consideration. In the study by Martinez‐Tellez et al. [32] dorsocervical uptake was predominantly observed in very young adults (mean age approximately 21 years), whereas in the cohort examined by Fletcher et al., women exhibiting dorsocervical uptake were older on average (mean age 27.8 years). Despite differences in sample size, age distribution, and study design, both investigations report convergent patterns consistent with a higher prevalence of dorsocervical BAT‐like glucose uptake in women. Collectively, these findings suggest that dorsocervical BAT‐like activity may be detectable in adult humans, particularly in women, while underscoring the need for further studies to clarify the anatomical identity, cellular composition, and physiological relevance of this depot.

**FIGURE 2:**
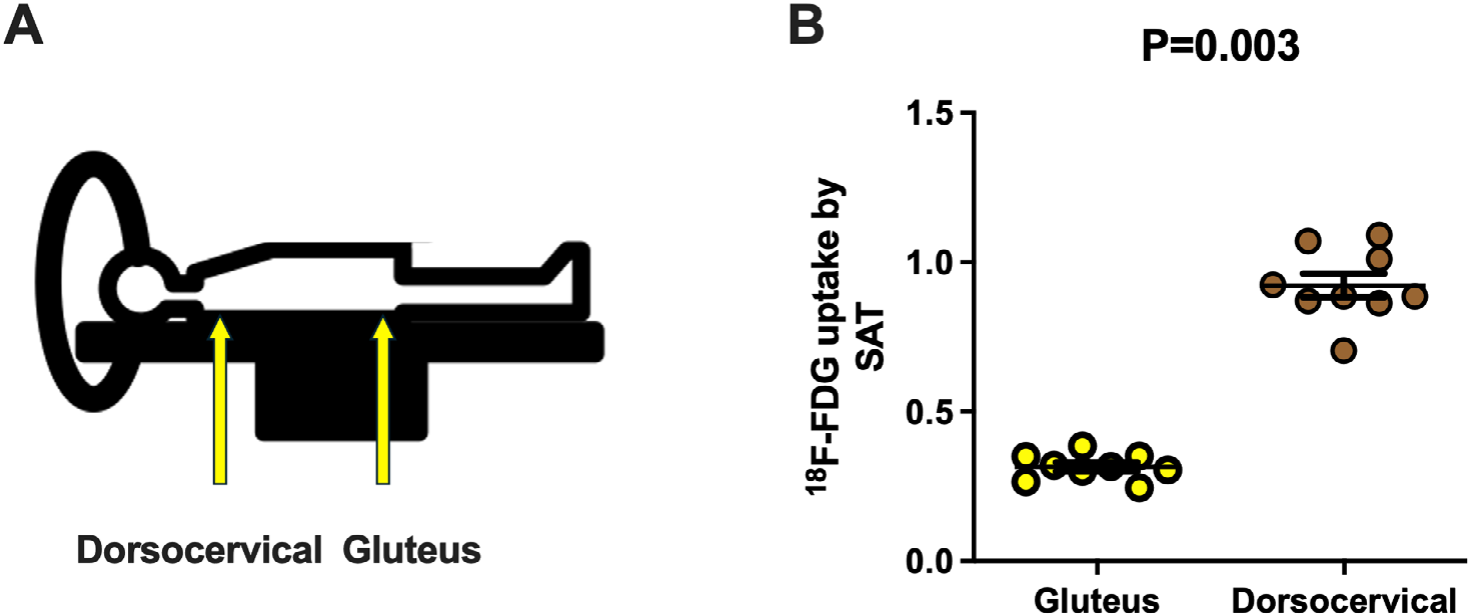
Cold‐induced ^18^F‐FDG uptake (Standardized uptake value peak (SUVpeak)) in the gluteal region and dorsocervical region, where body weight exerted pressure against the scanning bed, in the same nine male individuals. Panel A shows a human body on a PET‐CT scan, highlighting the areas where the regions of interest were drawn. Panel B shows the ^18^F‐FDG uptake in subcutaneous adipose tissue (SAT) at the gluteal and dorsocervical areas from the same participants. The P‐value was obtained from one‐way analyses of variance obtained using SPSS. AT, Adipose tissue; SAT, Subcutaneous adipose tissue.

One possible explanation for the higher glucose uptake observed in the dorsocervical region of young individuals, compared to other adipose tissue depots, is the susceptibility of this area to imaging artifacts. These artifacts may arise from factors such as body compression against the scanning bed and partial volume effects. To test this hypothesis, we analyzed whole‐body ^18^F‐FDG PET‐CT scans acquired in the Leiden University Medical Center (the Netherlands) [35] after a personalized cold exposure. In a cohort of nine males, we defined regions of interest (ROIs) in the subcutaneous adipose tissue of the dorsocervical region and the gluteal region. In both cases, the ROIs were placed on subcutaneous adipose tissue that was in direct contact with the scanning bed. Our analysis revealed that the standardized uptake value peak (SUVpeak) was 0.92 ± 0.12 in the dorsocervical region and 0.32 ± 0.04 in the gluteal region, indicating a 2.9‐fold higher glucose uptake in the dorsocervical area (see Figure 2). Based on these observations, we cautiously hypothesized that body pressure exerted on the subcutaneous adipose tissue during cold‐induced ^18^F‐FDG PET‐CT scanning may not fully explain the observed differences in glucose uptake, as the subcutaneous depots assessed are subjected to comparable pressure against the scanning bed. Nevertheless, these observations should be interpreted with caution, and further studies are required in which participants are positioned in the prone position, lying with the abdomen against the scanning bed after cold exposure, to more directly assess whether body positioning influences glucose uptake in these regions.

## 4 The Use of Single‐Cell Omics for Understanding the Composition of iBAT

In 1987, Kellman et al. [36] conducted a detailed study analyzing the tissue composition of the supraclavicular fossa in human corpses. They found that this area contains the subclavian vessels, the brachial plexus, the omohyoid and scalene muscles, BAT [37], and, to a lesser extent, lymph nodes and the posterior lung apex [36]. They described the supraclavicular *fossae* as a highly heterogeneous region composed of various tissues with extensive vascularization. As a result, obtaining human BAT biopsies from this area is particularly challenging. Nonetheless, some research groups have successfully obtained BAT biopsies from both patients and healthy adults. The most commonly used procedure involves performing a cold‐induced ^18^F‐FDG PET‐CT scan, followed by a surgical radioguided technique to obtain supraclavicular BAT [38]. However, this technique is highly invasive for participants, potentially risky [39], and poses significant challenges for researchers to optimize across different institutions. As a result, most human BAT biopsies collected so far in the field come from patients with a suspected type of cancer in the neck region or thyroid surgeries. This naturally biases BAT sample collection, as these patients are typically older adults (aged 50–65 years) and may have cancer, which could influence adipose tissue function [40]. Therefore, if the molecular presence of iBAT can be confirmed in adults, this could open the opportunity to perform subcutaneous BAT biopsies, which could substantially advance our understanding of the role of this tissue in metabolism.

Recent advances in single‐cell omics technologies, such as single‐cell and single‐nucleus RNA sequencing (scRNA‐seq and snRNA‐seq), spatial transcriptomics, and spatial proteomics/metabolomics, have significantly deepened our understanding of adipose tissue heterogeneity, cellular function, and intercellular communication [41]. Among these, snRNA‐seq has proven particularly valuable for profiling gene expression at the level of individual nuclei, especially in complex tissues like adipose tissue. Using snRNA‐seq, seven subpopulations of adipocytes in the subcutaneous adipose tissue over the neck and eight subpopulations of adipocytes in the deep neck adipose tissue in different participants have been identified [42, 43]. More detailed, in the deep neck adipose tissue, they were able to characterize a subpopulation termed H‐Ad‐3 adipocytes, which might represent a subpopulation of catabolic adipocytes that utilize chemical energy to dissipate heat in human adipose tissue. Based on this, they performed RNA‐seq on subcutaneous adipose tissue from 1099 participants/patients and found that the abundance of H‐Ad‐3 adipocytes was inversely correlated with human metabolic health parameters (age, % hemoglobin A1c [%HbA1c], BMI, fasting blood glucose, triglycerides, leptin, and weight). Additionally, in a sub‐cohort (*n* = 15), they found that the abundance of H‐Ad‐3 adipocytes was positively correlated with cold‐induced thermogenesis [42]. While this association suggests that H‐Ad‐3 adipocytes may be linked to thermogenic capacity, the implications for metabolic health remain unclear. As these findings are based on correlational analyses and do not establish causality, further targeted physiological and mechanistic studies are required to determine whether this adipocyte subpopulation plays a functional role in thermogenesis or metabolic regulation.

To better understand the role of adipocyte subpopulations in metabolic health, the authors also analyzed the cellular composition of visceral adipose tissue (VAT) and subcutaneous adipose tissue in 77 individuals with either a metabolically healthy obesity (MHO) or metabolically unhealthy obesity (MUO) phenotype [44]. Their analyses revealed marked differences in VAT adipocyte composition between MHO and MUO individuals, with widespread alterations across multiple cell populations, whereas subcutaneous adipose tissue composition remained largely unchanged. Notably, MHO individuals exhibited a higher proportion of mesothelial cells in VAT and a greater fraction of visceral adipocytes, suggesting preserved adipocyte hyperplasia. These observations are relevant in the context of thermogenic adipose tissue biology, as both VAT and BAT are highly vascularized and innervated depots whose metabolic activity is strongly influenced by cellular composition, immune cell recruitment, and adipocyte turnover. Consistent with this concept, we observed that metabolically healthy overweight‐obesity (MHOO) individuals displayed higher cold‐induced glucose uptake in BAT and the dorsocervical region, greater meal‐induced and cold‐induced thermogenesis, and lower VAT mass compared with MUOO individuals [33]. Together, these findings suggest that a metabolically healthy phenotype may be associated with shared features across adipose depots, including preserved adipocyte plasticity and a supportive cellular microenvironment, rather than depot‐specific effects alone. In addition, the identification of a female‐specific population of highly secretory, anti‐adipogenic progenitor cells in VAT further supports the notion that sex‐specific cellular programs may contribute to coordinated regulation of adipose tissue function across depots [44]. Collectively, these data raise the hypothesis that dorsocervical adipose tissue, similar to VAT, may harbor a distinct cellular and immune landscape that supports enhanced metabolic activity, although direct evidence for this link remains to be established.

The studies described above demonstrated that adipose tissue within the same anatomical region can exhibit distinct adipocyte composition depending on whether it is located subcutaneously, viscerally, or intramuscularly. However, it remains unclear whether adipocyte subpopulations differ between BAT and WAT in humans. To investigate this, Palani et al. [45] applied snRNA‐seq to various adipose tissue depots after differentiation, including VAT (collected during gallbladder surgery; *n* = 3), perirenal adipose tissue (obtained during nephrectomy; *n* = 3), abdominal subcutaneous adipose tissue (sampled using the Bergström needle biopsy; *n* = 4), and supraclavicular BAT (collected during surgery in patients with suspected neck cancer; *n* = 4). Although all the samples were obtained from different individuals (mean: 48.2 years old, range 29–62 years old), introducing inter‐individual variability, the study revealed that two distinct cell types arise from a common progenitor at the onset of differentiation in human BAT and WAT: a classical adipogenic cell type and an alternative cell type characterized by the expression of extracellular matrix‐secreted and developmental genes, which they termed Wnt‐regulated adipose tissue‐resident (SWAT) cells. These findings suggest a dynamic balance between these two cell fates, each playing complementary roles in adipose tissue architecture and function in humans.

Importantly, similar paradigm shifts have occurred in other tissues. In skeletal muscle, single‐cell approaches have shown that the traditional classification into type I and type II fibers is overly simplistic, revealing instead a spectrum of fiber subtypes defined by myosin heavy chain expression [46]. By analogy, BAT may likewise encompass a range of adipocyte states that cannot be fully captured by binary BAT versus WAT classifications. In this context, it is plausible that the dorsocervical region in humans harbors a heterogeneous adipocyte population, potentially enriched in beige‐like cells. This hypothesis is supported by developmental evidence indicating that this region is predominantly BAT during childhood, along with observations suggesting its persistence into early adulthood or reactivation under certain pathological conditions. Together, these considerations highlight the importance of systematically investigating the cellular composition of dorsocervical adipose tissue to determine whether it represents a classical brown, beige, or mixed adipose depot in adult humans.

## 5 Future Directions

Future studies should focus on defining the cellular identity and functional relevance of dorsocervical subcutaneous adipose tissue in humans. Although imaging studies suggest BAT‐like activity in this region, ^18^F‐FDG PET‐CT scans alone cannot determine adipocyte phenotype or physiological function. Improved imaging protocols, including cold‐induced ^18^F‐FDG PET‐CT scans performed with participants in the prone position, may help exclude confounding effects of body pressure and better characterize regional glucose uptake. Additionally, future studies should incorporate PET techniques that enable direct quantification of thermogenic activity in vivo, such as those using ^15^O_2_, ^11^C‐acetate, or H_2_O tracers. Direct tissue interrogation will be essential. Targeted biopsies of dorsocervical adipose tissue, analyzed alongside supraclavicular BAT and abdominal WAT, are needed to determine whether this depot contains classical BAT, beige adipocytes, or a mixed population. Initial studies may focus on young women, but validation across sexes, age groups, and metabolic phenotypes is required. Single‐cell and spatial omics approaches will be critical for resolving the molecular composition of this depot, identifying relevant adipocyte and non‐adipocyte cell populations, and determining how different interventions modify adipocyte cell populations. These analyses should be complemented by functional assays, including β‐adrenergic stimulation, lipolysis, mitochondrial respiration, and substrate utilization, to assess whether dorsocervical adipose tissue exhibits intrinsic BAT‐like activity. Intervention studies involving cold exposure, exercise, or pharmacological stimulation may clarify whether this depot retains an enhanced browning capacity, consistent with its BAT identity during early life. In parallel, in vitro studies comparing the secretory profile of dorsocervical adipose tissue with supraclavicular BAT may reveal endocrine or paracrine functions independent of thermogenesis.

## 6 Conclusions

**FIGURE 3:**
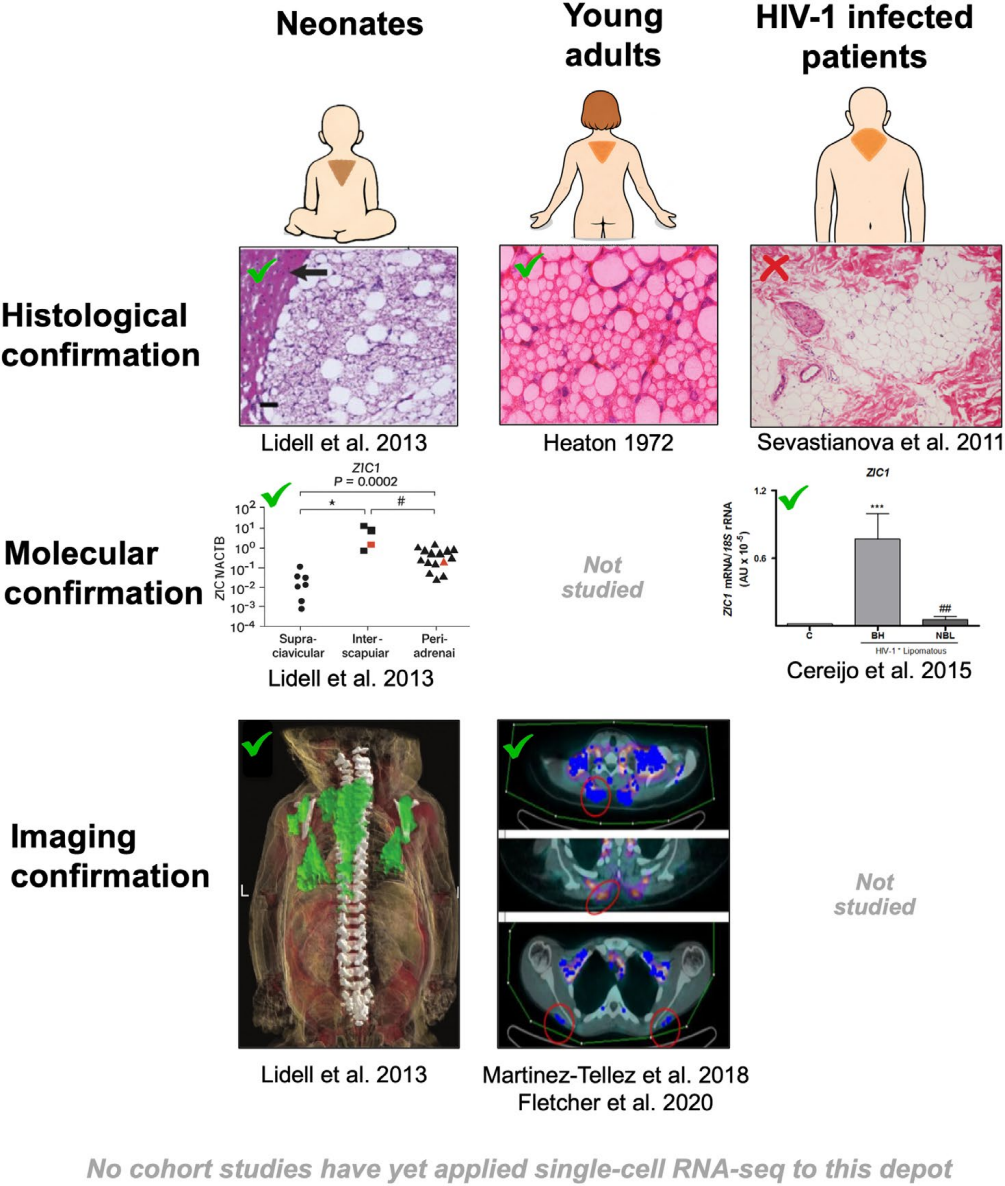
Evidence supporting the presence of interscapular brown adipose tissue (iBAT) in humans across the lifespan. Histological, molecular, and imaging evidence for iBAT is summarized across different populations. Lidell et al. [9] provided direct histological, molecular, and imaging evidence for the presence of iBAT in neonates. Heaton reported histological identification of iBAT in young adults [13]. In adults, Martinez‐Tellez et al. [32] and Fletcher et al. [34] demonstrated higher glucose uptake in the dorsocervical area using cold‐induced ^18^F‐FDG PET‐CT imaging. In people living with HIV‐1, Sevastianova et al. [27] were unable to histologically detect iBAT in the dorsocervical region, whereas Cereijo et al. [21]. reported molecular evidence based on the expression of UCP1 (data not shown in this figure) and ZIC1, a gene enriched in iBAT. Overall, future studies using advanced tracers to assess thermogenic activity in vivo (e.g., [^15^O]H_2_O or [^15^O]O_2_) are warranted. The colors in the histology figure from Heaton were added using AI, since the original image was in black and white.

In conclusion, subcutaneous adipose tissue in the dorsocervical region has been conclusively identified as iBAT in human neonates through gene expression analyses and histological characterization. In adults, the presence of iBAT tissue was postulated by Heaton [13] based on histological observations, although definitive molecular confirmation remains lacking. Evidence from HIV‐1–infected individuals indicates increased expression of BAT‐associated markers in the dorsocervical region; however, histological analyses have not consistently confirmed the presence of iBAT in this population (see Figure 3). In addition, two independent studies using cold‐induced ^18^F‐FDG PET‐CT imaging have reported elevated glucose uptake in the dorsocervical area, with a higher prevalence observed in women. However, targeted studies combining advanced imaging approaches with tissue biopsies are required to determine whether dorsocervical adipose tissue in adults exhibits a brown or beige adipose tissue–like molecular signature and whether it is thermogenically active.

## Author Contributions

J.S.‐G., S.R.‐C., A.C.‐P., A.B.‐R., C.W., P.C.N.R., F.A.M. and T.R.: Writing – review and editing; conceptualization. B.M.‐T.: Writing original draft – review and editing; conceptualization; funding acquisition.

## Funding

Grants PID2022‐141442OA‐I00 funded by MICIU/AEI/10.13039/501100011033, by ERDF/EU and by ESF+ (FPI position to JSG). TR and BMT are recipients of Ramón y Cajal grant (Grant RYC2022‐035807‐I and RYC2022‐036473‐I, respectively) by MCIN/AEI/10.13039/501100011033 and by ESF+. This project was partially funded by EASO‐New Clinical Investigator Award 2024, the EFSD‐Rising Star 2024, and the Acta Physiologica Rising Star Award 2024.

## Conflicts of Interest

The authors declare no conflicts of interest.

## Data Availability

Data sharing is not applicable to this article as no new data were created or analyzed in this study.
